# Renoprotective effects of antioxidants against cisplatin nephrotoxicity

**Published:** 2014-07-01

**Authors:** Shabnam Hajian, Mahmoud Rafieian-Kopaei, Hamid Nasri

**Affiliations:** ^1^Medical Plants Research Center, Shahrekord University of Medical Sciences, Shahrekord, Iran; ^2^Department of Nephrology, Isfahan University of Medical Sciences, Isfahan, Iran

**Keywords:** Cisplatin, Nephrotoxicity, Antioxidant

## Abstract

Nephrotoxicity is the major limitation for the clinical use of cisplatin as an anti-tumoural drug. Intracellular effects of cisplatin cause tubular damage and tubular dysfunction with sodium, potassium, and magnesium wasting. Renoperotective strategies against cisplatin are classified on 8 targets: 1) Decrease of cisplatin uptake by renal cell, 2) Inhibition of cisplatin metabolism, 3) Blocking cell death pathways, 4) Cyclin-dependent kinase inhibitors, 5) Pharmacologic, molecular, and genetic blockade of p53, 6) Inhibition of specific Mitogen-activated protein kinase, 7) Antioxidants usage for renoprotection against cisplatin injury and inhibit of oxidative stress, 8) Suppress of inflammation. The oxidation reactions can produce free radicals, which start chain reactions and subsequently can cause a large number of diseases in humans. Antioxidant from natural products have attracted the physicians’ attentions, nowadays. The natural product antioxidants detoxify reactive oxygen species (ROS) in kidneys, without affecting the anticancer efficacy of cisplatin. Hence, antioxidants have potential therapeutic applications.

**Implication for health policy/practice/research/medical education:**

Antioxidant from natural products have attracted nowadays. The natural product antioxidants detoxify reactive oxygen species (ROS) in kidneys, without affecting the anticancer efficacy of cisplatin thus antioxidants could have potential therapeutic applications.

## Introduction

Cisplatin is a major chemotherapy drug for the treatment of solid tumors. It leads to accumulation of platinum within the kidney and disturb renal tubular tissue and function. However its side effects in normal tissues and organs, notably nephrotoxicity in the kidneys, cause dose limiting in therapeutics. Side effects in normal tissues, including neurotoxicity, ototoxicity, nausea and vomiting are liming factors of cisplatin use (1). Mechanism of cisplatin nephrotoxicity in the kidney revealed: 1) a decrease in renal blood flow and glomerular filtration rate, 2) tubular necrosis/apoptosis, 3) increased lipid peroxidation and decreased endogenous antioxidant systems, 4) increased expression of inflammation markers and 5) increased activity of the apoptosis executioner caspase-3. Renoprotective approaches are being discovered, but the protective effects are mostly unknown, recommending the need for combinatorial strategies whilst it is unclear whether these approaches would limit the anticancer effects of cisplatin in tumors (2).

## Renoprotective strategies against cisplatin therapy

These approaches can be generally classified on 8 targets: 1) Decrease of cisplatin uptake by renal cells, 2) Inhibition of cisplatin metabolism, 3) Blocking cell death pathways, 4) Cyclin-dependent kinase inhibitors, 5) Pharmacologic, molecular, and genetic blockade of p53, 6) Inhibition of specific Mitogen-activated protein kinases (MAPKs), 7) Antioxidants usage for renoprotection against cisplatin injury and inhibit of oxidative stress, 8) Suppress of inflammation (3-6).

Cisplatin is used for the treatment of testicular, head and neck, ovarian, cervical, nonsmall cell lung carcinoma, and many other types of cancer. The molecular mechanism of anticancer of cisplatin is bound to DNA, leading to the formation of inter- and intra-strand cross-links which activate several signal transduction pathways, involving p53, p73, and MAPK, and reach the highest activation of apoptosis. DNA damage-mediated apoptotic signals, can be attenuated, and the resistance that occurs is a major limitation of cisplatin-based therapeutics (7-10).

## Cisplatin nephrotoxicity

Nephrotoxicity of cisplatin is often seen after 10 days and complications include lower glomerular filtration rate, higher serum creatinine, and reduced serum magnesium or potassium levels. Cisplatin-induced kidney injury is associated with increased kidney vascular resistance and histological damage to proximal tubular cells resulting in decreased blood flow and ischemic injury of the kidneys, contributing to a decline in glomerular filtration rate. The cytotoxicity of cisplatin has multiple intracellular effects, including regulating genes, causing direct cytotoxicity with reactive oxygen species (ROS), activating MAPKs, apoptosis, stimulating inflammation and fibrogenesis. These events cause tubular damage and tubular dysfunction with sodium, potassium, and magnesium wasting. It is due to a combination of cell membrane peroxidation, mitochondrial dysfunction, inhibition of protein synthesis, and DNA injury. These events conclude the loss of renal function during cisplatin nephrotoxicity, and acute renal failure (11,12).

## The cellular pathway of cisplatin injury of kidney cells

Cisplatin increases ROS production via the disrupted respiratory chain and induce mitochondrial dysfunction. Cisplatin induces ROS formation in the microsomes via the cytochrome P450 system. This drug causes break down of nuclear and mitochondrial DNA and production of ROS leads to activation of both mitochondrial and non-mitochondrial pathways of apoptosis and necrosis. ROS in renal epithelial cells reduce the activity of antioxidant enzymes and deplete intracellular concentrations of glutathione (GSH). Nephrotoxicity of cisplatin is amassing of it in the tubular epithelial cells of proximal kidney tubule and is characterized by morphological destruction of intracellular organelles and cellular necrosis (13,14). Oxidative stress plays an important role in development of kidney disease including glomerular injury or promoting hypertension and atherosclerosis or kidney ischemia. In addition, it consequences decrease of natural cell antioxidant capacity or increase in quantity of ROS in kidney. Numerous studies have reported toxic effects of cisplatin induced nephrotoxicity. A growing amount of results provide evidence that toxic drugs are capable of interacting with nuclear proteins and deoxyribonucleic acid (DNA) causing oxidative deterioration of biological macromolecules (15,16).

Recent studies have focused on the role of antioxidants in cisplatin toxicity. Also the oxidative stress induced cisplatin in the kidney was partially inhibited by antioxidant therapy using antioxidants such as vitamin C or E, flavonoids, superoxide dismutase, glutathione and selenium, as well as plants antioxidants (17).

The antioxidants are bioactive molecules which are capable of decreasing or preventing the oxidation of substrate molecules. The oxidation reactions can produce free radicals, which start chain reactions and subsequently can cause a large number of diseases in humans (18). Antioxidant from natural products have attracted a lot of attentions, nowadays. The natural product antioxidants may detoxify ROS in kidneys, without affecting the anticancer efficacy of cisplatin. Hence, antioxidants have potential therapeutic applications.

Antioxidant compounds remove free radical intermediates, and inhibit other oxidation reactions by being oxidized themselves (18-22). Antioxidants trap free radicals, terminating the chain reaction by chelating metal ion and preventing the reaction with ROS or by chelating metal and protecting against metal toxicity (23). Chelating metal are capable of binding to toxic metal ions to form complex structures which are easily excreted from the body removing them from intracellular or extracellular spaces. The concept of cisplatin therapeutic is based on simple coordination of herbal plants, evolution of an ideal chelator and chelation therapy that completely removes specific toxic metal from desired site in the body which involves an integrated drug design approach (24-26).

Some herbal medicines have also been shown to protect kidney injury. Natural products from medical plants have capacity to ameliorate oxidative stress. Phytochemicals play an important role as natural antioxidants and immunomodulators (27-31).

## Natural antioxidants

Antioxidant compounds including tocopherols, flavonoids, carotenoids, and phenolic compounds can inhibit Fe3+ induced oxidation and scavenge free radicals. Also they act as reductants spices and are used in medicine. It is notable that phenolic compounds have strong H-donating activity. Antioxidants protect biomolecules from free radical damage induced by both ROS and reactive nitrogen species (RNS) (32,33).

The plant phenolic antioxidants are divided into four general groups: phenolic diterpenes (carnosol and carnosic acid), phenolic acids (gallic, protocatechuic, caffeic, and rosmarinic acids), flavonoids (quercetin and catechin), and volatile oils (menthol). Generally phenolic acids act as antioxidants by trapping free radicals and flavonoids can scavenge free radicals and chelate metals as well and biological aspects of antioxidants are particularly related to their chelating properties.

Also flavonoids with multiple hydroxyl groups are more effective antioxidants than the other ones with only one (34-36).

In agreement with prior investigations, we found that cisplatin induced tubular injury, increased inflammatory cell infiltration, oxidative/nitrative stress and impaired renal function. Cisplatin-induced tubular damage and nephropathy revealed by mitochondria injury and oxidant stress (37).

We recently conducted another investigation on rat model of cisplatin nephrotoxicity and observed that in addition to antioxidants, losartan may prevent cisplatin nephrotoxicity in males. We also recently observed that, vitamin E and vitamin C are chemoprotective agents against cisplatin nephrotoxicity. Also Selenium is a component of the antioxidant for enzymes glutathione peroxidase and thioredoxin reductase (38,39). Notably, magnesium supplementation during chemotherapy with cisplatin/paclitaxel is a nephroprotective management with no reduction of antitumor efficacy (40). We noticed that, erythropoietin is capable of protecting renal injury, and may lead to different responses against cisplatin-kidney injury in rat model. We found that, treatment by recombinant human erythropoietin (Eprex) abolished changes in blood urea nitrogen and creatinine levels in rat model (41,42). Also oxytocin ameliorates cisplatin-induced nephrotoxicity (43).

Investigations have revealed that crude caffeine did possess hydrophilic antioxidant activity and lipophilic antioxidant activity, and its administration has led to the inhibition of cyclooxygenase-2 enzyme. Also, caffeine is able to recover toxicity of cisplatin (44,45).

We also observed that cisplatin lonely increased kidney damage significantly, but the injury induced by combination of cisplatin and nitric oxide synthase can inhibit the damage of the kidney (46).

Antioxidants are different substances such as vitamin C, vitamin E, carotenoids, minerals such as selenium and manganese, as well as glutathione, coenzyme Q10, lipoic acid, phytoestrogens, flavonoids, phenols, and polyphenols which largely prevent the functional and structural lesions in tubular of kidney. These agents can prevent cisplatin induce mitochondrial production of oxidants which are the key to its ability to injure mitochondria (37-47).

In case of cisplatin toxicity, due to excess production of highly reactive free radicals, an imbalance occurs in the oxidant-antioxidant status leading to depletion in the activities of antioxidants as well as an elevation in lipid peroxidation. ROS like hydrogen peroxide, superoxide and hydroxyl radicals generated under normal metabolic conditions are generally detoxified by the activities of antioxidants like reduced glutathione, superoxide dismutase (SOD) and catalase (CAT). The activities of key enzymatic antioxidants like SOD, CAT, glutathione peroxidase (GPx) and glutathione S-transferase (GST) were found decreased in cisplatin induced nephrotoxic (48).

## Authors’ contributions

All authors contributed to the paper equally.

## Conflict of interests

The authors declared no competing interests.

## Ethical considerations

Ethical issues (including plagiarism, misconduct, data fabrication, falsification, double publication or submission, redundancy) have been completely observed by the authors.

## Funding/Support

None.
